# Access to Barrier Perches Improves Behavior Repertoire in Broilers

**DOI:** 10.1371/journal.pone.0029826

**Published:** 2012-01-27

**Authors:** Beth A. Ventura, Frank Siewerdt, Inma Estevez

**Affiliations:** 1 Department of Animal and Avian Sciences, University of Maryland, College Park, Maryland, United States of America; 2 IKERBASQUE, Basque Foundation for Science, Alameda Urquijo, Spain; 3 Neiker Tecnalia, Arkaute Agrifood Campus, Vitoria-Gasteiz, Spain; Liverpool John Moores University, United Kingdom

## Abstract

Restriction of behavioral opportunities and uneven use of space are considerable welfare concerns in modern broiler production, particularly when birds are kept at high densities. We hypothesized that increased environmental complexity by provision of barrier perches would help address these issues by encouraging perching and enhancing use of the pen space across a range of stocking densities. 2,088 day-old broiler chicks were randomly assigned to one of the following barrier and density treatment combinations over four replications: simple barrier, complex barrier, or control (no barrier) and low (8 birds/m^2^), moderate (13 birds/m^2^), or high (18 birds/m^2^) density. Data were collected on focal birds via instantaneous scan sampling from 2 to 6 weeks of age. Mean estimates per pen for percent of observations seen performing each behavior, as well as percent of observations in the pen periphery vs. center, were quantified and submitted to an analysis of variance with week as the repeated measure. Barrier perches, density and age affected the behavioral time budget of broilers. Both simple and complex barrier perches effectively stimulated high perching rates. Aggression and disturbances were lower in both barrier treatments compared to controls (P<0.05). Increasing density to 18 birds/m^2^ compared to the lower densities suppressed activity levels, with lower foraging (P<0.005), decreased perching (P<0.0001) and increased sitting (P = 0.001) earlier in the rearing period. Disturbances also increased at higher densities (P<0.05). Use of the central pen area was higher in simple barrier pens compared to controls (P<0.001), while increasing density above 8 birds/m^2^ suppressed use of the central space (P<0.05). This work confirms some negative effects of increasing density and suggests that barrier perches have the potential to improve broiler welfare by encouraging activity (notably by providing accessible opportunities to perch), decreasing aggression and disturbances, and promoting more even distribution of birds throughout the pen space.

## Introduction

Broiler production has risen exponentially since the 1940s as commercial operations have become vertically integrated and confinement has grown increasingly intensive [1]. While such expansion has undoubtedly resulted in significant economic progress, it has also prompted concerns regarding repercussions on poultry welfare. Behavioral restriction and uneven use of space, especially in high density rearing scenarios, are of particular issue.

Broilers in large-scale production tend to be reared in environments of low complexity, where required travel and foraging effort is low and bird movement and activity is limited [2], [3], [4]. It is in such contexts of low activity that restriction of natural behavior is most likely to occur [5]. Compounding the issue is broiler inactivity, potentially influenced by two factors: lameness caused by increased growth rate and body weight [6] and restricted opportunity for movement due to high stocking density levels [2], [7].

The effects of stocking broilers at high densities are reflected in a reduction of behavioral expression. Locomotion and distance traveled have consistently been reported to decline as density increases [2], [8], [9], perhaps because more birds per unit area create a barrier effect by hampering bird dispersion throughout the pen [2], [7]. Birds also spend less time resting at higher densities due to a resultant increase in disturbances [8], [10]. Further, increases in density appear to have a suppressive effect on behaviors like scratching and walking, though this may be due to indirect effects of increasing density – like a decline in litter quality – rather than to a lack of space [11].

Adequate physical space is required for an animal to express the full complement of behaviors intrinsic to its biology. However, individual broilers in flocks do not utilize the entire space available to them in experimental [9] or commercial facilities [12]. Rather, they tend to cluster around the periphery, other objects or among themselves in a reflection of anti-predation mechanisms [7], [13], [14]. Such uneven use of the pen can cause a host of welfare issues. Aggregations around the periphery create an underused central area, providing prime space for agonistic interactions [10], [15]. Individuals entering or leaving such clusters disrupt the resting intervals of birds already in the group [10]. Finally, as temperatures increase reduced airflow may subject individuals within these aggregations to heat stress [16], [17].

Given these issues, we assert the importance of seeking avenues to promote maximal use of space along with an increased range of behavioral expression. Increasing the complexity of the environment by adding enrichment can have a substantial impact on broiler welfare [12], [18], [19], in part by providing substrate upon which previously restricted behaviors can be expressed [20]. Perching serves as a good example in intensive broiler production, as past attempts to stimulate perching in broilers have not yielded promising results [21]–[23]. However, since higher perching rates have been observed with application of wooden barriers [19], it is likely that perching is still a motivated behavior in broilers. Providing barrier perches – which are grounded lower than other types of perches and hence more adapted to the heavier nature of modern broilers – may provide enhanced opportunities to express a wider variety of natural behaviors. Barrier perches may also be expected to function similarly to cover panels [13] by attracting birds away from the pen periphery in a way that achieves improved bird dispersion throughout the pen space.

It is currently unknown how barrier perches may manifest these effects when birds are stocked at industry-level densities. Our objective was to explore the effects of providing barrier perches at varying densities on the behavior repertoire and use of space in broiler chickens.

## Materials and Methods

### Animals and experimental setup

A total of 2474 day-old, straight run broiler chicks (Ross 308) were obtained from a commercial hatchery and placed into experimental pens on day 1. Each experimental pen had an area of 4.46 m^2^ and was bedded with approximately 5 cm of wood shavings. Feed and water were provided *ad libitum*. A large tubular feeder and one line of nipple drinkers were positioned along one side of each pen before chick placement. Feed was also provided in two shallow feed trays during the first two weeks. The feeding program consisted of a standard three-phase commercial diet. Feeders and drinkers were partially blocked in order to maintain equal resource access per bird regardless of density treatment. The lighting program used was 24L∶0D from day 0–2 and 14L∶10D for the remainder of the experiment. Temperature and ventilation practices were standard. Further management details are provided elsewhere [24].

On day 1, ten birds per pen (360 birds total) were randomly designated as focal birds and tagged on both sides of the neck for identification using the Swiftack Poultry Identification System (Heartland Animal Health, Inc., Fair Play, MO). This tagging protocol allows for swift and permanent identification of individuals without negatively affecting behavior or welfare [13], [21], [23].

The birds were randomly divided into 36 groups and assigned to one of three barrier treatments at one of three stocking densities (3×3 factorial). Barrier-density combinations were replicated four times. Barrier treatments were: simple barrier, complex barrier, and no barriers (control). Simple barrier treatment pens contained three wooden barriers measuring 100 cm×15 cm×4 cm (length×height×width). Complex barrier treatment pens also contained three barriers of the same dimensions as those used in the simple barrier treatment, but two of the barriers had three additional ‘arms’ attached to one side, creating an ‘E’ shape when viewed from above. Each arm measured 20 cm×15 cm×4 cm (length×height×width). The bases of all barriers were bracketed for stability. Once placed in the pens containing 5 cm of wood shavings, the effective height of all barriers was 10 cm. All barriers were placed in a staggered setup in two rows between the food and water sources. See [24] for a schematic layout of the experimental pens.

Original experimental stocking densities were: low, moderate, and high, corresponding to 10 birds/m^2^ (45 birds/pen), 15 birds/m^2^ (67 birds/pen) and 20 birds/m^2^ (90 birds/pen), respectively. An unexpectedly high 7% mortality rate during the first week was attributed to poor chick quality, as the effect of treatment on first week mortality was nonsignificant. New lower stocking densities were calculated to 8, 13, and 18 birds/m^2^ (36, 58, and 80 birds/pen, respectively) and birds were redistributed on day 7 for the following reason: some pens had exceedingly high mortality rates to the extent that existing bird counts within those pens did not satisfy even the new lower density requirements. In these cases, care was taken to ensure that any birds added to pens to meet the required number had come from pens with the same treatment conditions. This research protocol (R-08-01) was approved by the Institutional Animal Care and Use Committee at the University of Maryland.

### Observations and data collection

Behavioral and spatial data were collected on focal birds via instantaneous scan sampling [25]. Recorded behaviors included: feeding, drinking, foraging, aggression, disturbances, standing, sitting, walking, running, perching, preening, dust bathing, flapping, and other (adapted from [10], [18], see Table 1). Focal birds' locations in the pen were recorded simultaneously. Each of the 36 pens was visited in a random order; this cycle was repeated twice per day, four times per week from weeks 2 to 6, producing a total of 1440 pen scans for the study. Week 1 observations were omitted due to the early high mortality rate. Data collection was performed between 8:00 h and 13:00 h to reduce variability attributable to diurnal behavior patterns [26]. Observations were conducted by the same researcher and lasted approximately 2–5 minutes per pen, depending on how quickly focal birds were located. Intervals between scans lasted only as long as it took the researcher to move to the next pen for observation. Data were recorded on a tablet PC (Toshiba, Inc.) using the Chickitizer program, (v.4. University of Maryland, College Park, Maryland, USA). This software allows each bird's location to be digitized into an XY coordinate system and recorded in conjunction with the identity and behavior of each particular focal bird at that point in time. Data collection was facilitated with the use of a numbered 7×10 grid coordinate system that was marked on all pen walls. Each cell in the grid measured approximately 26.14×24.40 cm.

**Table 1:** Experimental behavioral ethogram.

**Appetitive Behaviors**	
Feeding	Bird is located next to feeder and has its beak inside the feeder.
Drinking	Bird's head is raised toward nipple drinkers and is either attempting to or is currently contacting its beak with the drinker.
Foraging	Bird is pecking or scratching at the ground.
**Activity Behaviors**	
Sitting	Bird has ceased locomotion and its breast is in contact with the ground. Eyes may or may not be closed.
Standing	Bird maintains upright position on motionless, extended legs.
Walking	Relatively low-speed displacement of bird on the ground in which the propulsive force is derived from the action of the legs.
Running	Higher speed displacement of bird on the ground in which the propulsive force is derived from the action of the legs.
Perching	Bird's feet are grasping the barrier and bird is not locomoting. Breast of bird may or may not be in contact with barrier.
Dust bathing	Bird is lying on the ground and tossing dirt onto its back/wings by ruffling and shaking its feathers.
Flapping	Bird is in an upright position and extends its wings repeatedly.
Preening	Bird is using its beak to peck, stroke or comb plumage.
**Aggression** 1	
Chase	One bird runs at least three steps after another bird.
Fight	Two birds are standing facing each other with heads and necks raised to the same level. One bird delivers more than two vigorous kicks at opponent. Pecks may or may not be observed.
Leap	Two birds face each other; one or both jump without extending legs toward other bird.
Peck	Face-to-face encounter in which one bird raises its head and directs vigorous pecks toward another bird.
Standoff	Two birds facing each other with heads at same level for more than two seconds.
Threat	Bird stands with raised feathers and erect neck while opponent holds its head at lowered level.
**Disturbance**	Another bird makes physical contact with resting focal bird, causing it to readjust itself or stand.
**Other**	Any behavior not belonging to the previous categories was recorded under this label.

1 Observations in the categories “chase,” “fight,” “leap,” “peck,” “standoff,” and “threat” were recorded and analyzed under the “aggression” label.

Use of space was quantified by calculating the proportion of observations in the central vs. peripheral areas of the pen. The periphery was defined as the area within 25 cm of the pen walls (coinciding with approximately 1 cell width in the grid) and the remaining area was considered the central area [13]. Relative areas of the center and periphery were standardized by dividing the percent observations in either zone by the area of that zone before proceeding with analysis.

### Statistical analysis

Data that met analysis of variance (ANOVA) assumptions were analyzed using a model with barrier and density as fixed factors in a 3 by 3 factorial arrangement and week of age as the repeated measure unless otherwise stated. Analyses were performed using Statistical Analysis System v 9.1.3 [27] and significance was set at α = 0.05. P values above this threshold indicate non-significance and are not reported here.

#### Behavioral scans

The proportion of observations that birds were seen performing each behavior each day was determined for each focal bird and averaged across focal birds and days to obtain weekly pen means for analysis. Residuals for the behaviors feed, drink, sit, stand, walk, and preen met normality assumptions so these variables were analyzed with an ANOVA with week as the repeated measure and pen designated as the experimental unit. The perch behavior was analyzed with the same model after control pens were removed from the data set since it was not possible to observe perching in the control treatment. Data on the variables forage, aggress, disturb, and run were averaged across weeks to obtain overall pen means for the entire study period, which were then analyzed under a similar model except that repeated measures were not included. The flapping, dust bathing, and other behaviors occurred so infrequently that they were not included in the analysis. Tukey's HSD was used for all means comparisons [28].

#### Space use

The percentage of observed locations in the center vs. periphery was calculated for each focal bird and then averaged across focals within each pen to obtain weekly percent means per pen for analysis. As percent of observations in the periphery vs. center is complementary, only percent of observations in the central area is reported here. Mean percent of observations in the central area was analyzed with an ANOVA with repeated measures over weeks.

## Results

Interactions between factors were not significant unless otherwise stated.

### Appetitive behaviors

Stocking density did not affect the proportion of time birds were observed feeding or drinking. However, feeding and drinking were affected by both age (feeding: F_4,108_ = 21.55, P<0.0001 and drinking: F_4,108_ = 8.23, P<0.0001, Table 2) and barrier (feeding: F_2,27_ = 9.83, P<0.001 and drinking: F_2,27_ = 8.89, P = 0.001, Table 3). Feeding was observed less often in the complex barrier treatment versus the control pens (P<0.001) and was highest during the second week, generally declining thereafter (P<0.0001). Drinking was less frequently observed in birds in the simple barrier pens compared to controls (P<0.001) and was lowest during the second week of age (P<0.05). In contrast, the proportion of observations of foraging was not affected by barrier treatment but was affected by density (F_2,26_ = 8.90, P = 0.001; Table 4) such that the proportion of time that broilers spent foraging was significantly less for 18 birds/m^2^ compared to 8 or 13 birds/m^2^ (P<0.0001 for both comparisons).

**Table 2:** Behaviors^1^ (mean %) affected by age.

	Age (weeks)					Age effect^2^	
Behavior	2	3	4	5	6	F value	P value
Feeding	14.3±0.6^a^	9.9±0.6^b^	7.8±0.6^bc^	8.2±0.6^bc^	7.4±0.6^c^	21.55	<0.0001
Drinking	4.5±0.6^b^	6.7±0.5^a^	8.4±0.6^a^	7.1±0.5^a^	8.4±0.5^a^	8.23	<0.0001
Standing	5.3±0.7^d^	8.7±0.7^c^	14.3±0.7^a^	10.3±0.7^bc^	12.0±0.7^ab^	26.19	<0.0001
Walking	6.2±0.5^a^	5.9±0.5^ab^	7.2±0.5^a^	4.2±0.5^b^	4.2±0.5^b^	8.23	<0.0001
Preening	2.9±0.4^b^	6.3±0.4^a^	4.0±0.4^b^	3.4±0.4^b^	2.7±0.4^b^	13.44	<0.0001

**Figure 1:**
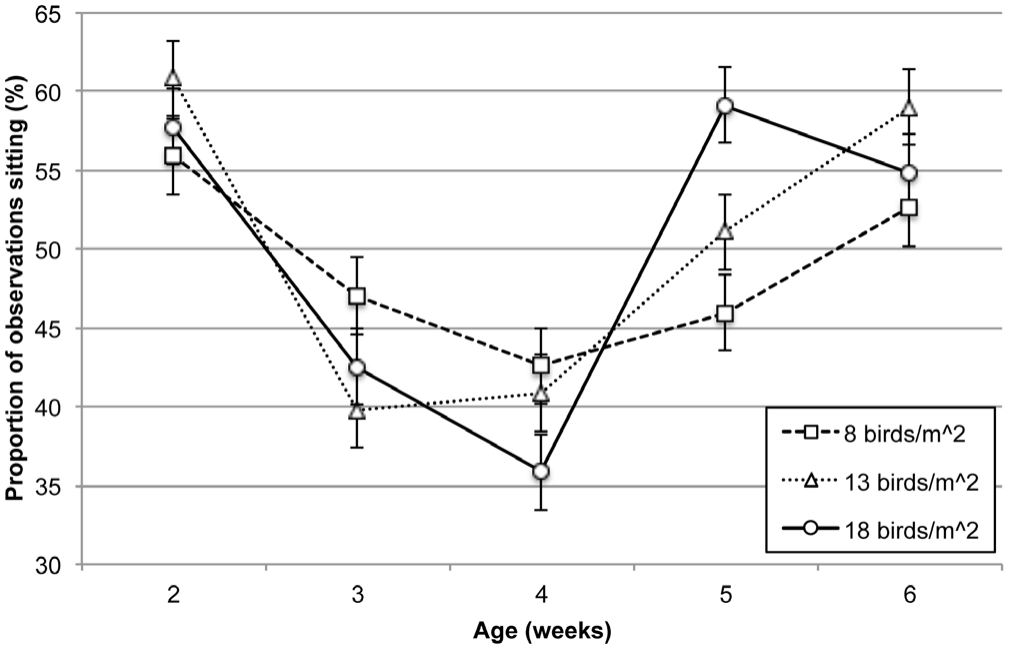
Density by age interaction effect of mean percent sitting (LSM ± SEM).

**Figure 2:**
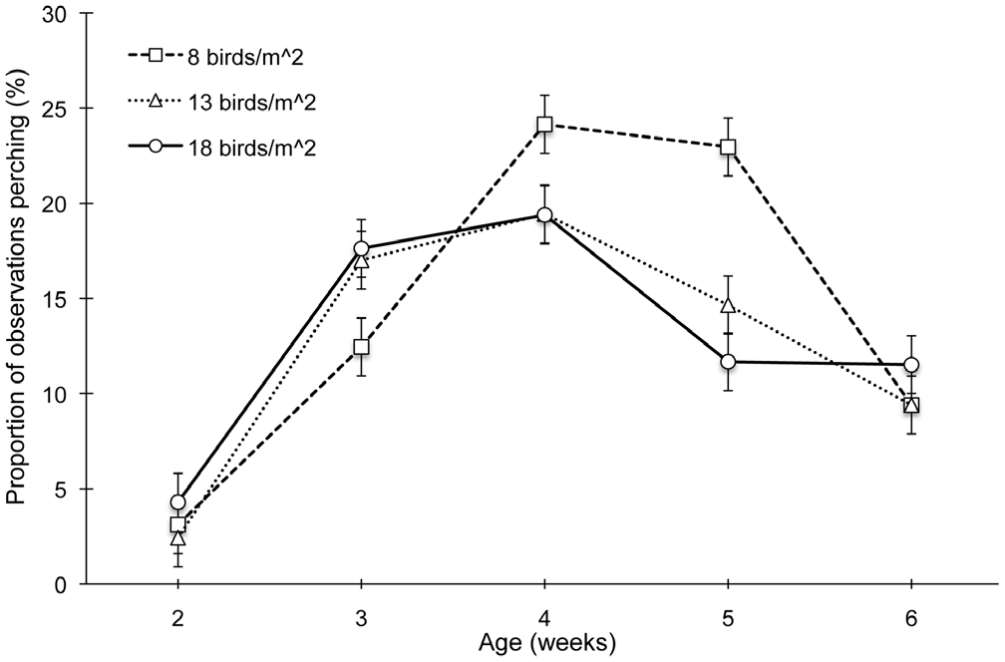
Density by age interaction effect on mean percent perching (LSM ± SEM).

1 Values are LSM ± SEM. “Foraging,” “running,” “aggression” and “disturbance” data were pooled over weeks. “Sitting” and “perching” were affected by age×density (Figs. 1 & 2). “Central area use” was not affected by age (F_4,108_ = 1.00).

2 Mixed model repeated measures ANOVA, df = 4,108.

a Means within a row with different superscripts are significantly different (P<0.05) after Tukey's comparison.

**Table 3:** Behaviors^1^ (mean %) affected by barrier treatment.

	Barrier treatment			Barrier treatment effect^2^	
Behaviors	Control	Simple	Complex	F value	P value
Feeding	11.0±0.4^a^	9.5±0.5^ab^	8.0±0.4^b^	9.83	<0.0001
Drinking	8.3±0.4^a^	5.8±0.4^b^	7.0±0.4^ab^	8.89	0.001
Standing	12.5±0.5^a^	9.0±0.5^b^	8.9±0.5^b^	15.68	<0.0001
Running	1.0±0.1^a^	0.6±0.1^b^	0.7±0.2^ab^	4.17	0.027
Aggression	0.9±0.1^a^	0.3±0.1^b^	0.02±0.1^b^	12.30	0.0002
Central area use	34.0±0.8^b^	38.8±0.8^a^	36.4±0.8^ab^	8.46	0.001

**Figure 3:**
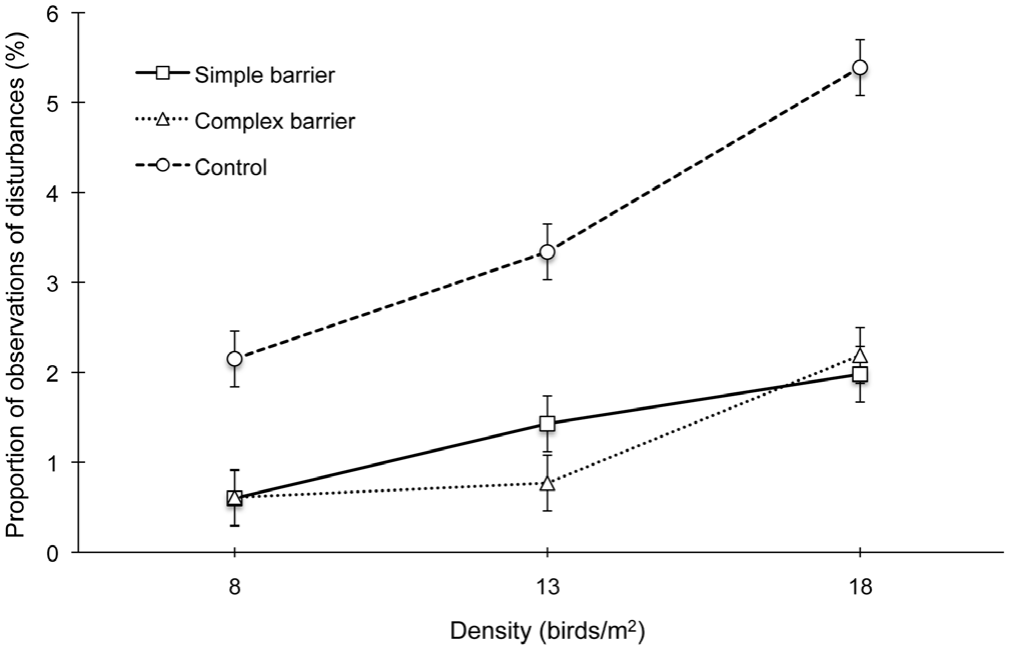
Barrier by density interaction effect on mean percent disturbances (LSM ± SEM).

1 Values are LSM ± SEM. “Foraging” (F_2,26_ = 0.38), “sitting” (F_2,27_ = 0.46), “walking” (F_2,27_ = 1.68), and “preening” (F_2,27_ = 2.54) were not affected by barrier treatment. “Perching” (F_1,18_ = 0.41) was not affected by barrier *type*. “Disturbance” was affected by barrier×density (Fig. 3).

2 Mixed model repeated measures ANOVA, df = 2,27, with the exception of running with df = 2,26 as running means were pooled across weeks.

a Means within a row with different superscripts are significantly different (P<0.05) after Tukey's comparison.

**Table 4:** Behaviors^1^ (mean %) affected by density treatment.

	Density treatment			Density treatment effect	
Behaviors	8 birds/m^2^	13 birds/m^2^	18 birds/m^2^	F value	P value
Foraging	2.7±0.3^a^	2.6±0.3^a^	1.3±0.3^b^	8.90	0.001
Walking	6.5±0.4^a^	5.1±0.4^b^	4.9±0.4^b^	6.45	0.005
Central area use	38.6±0.8^a^	35.2±0.8^b^	35.3±0.8^b^	5.70	0.009

1 Values are LSM ± SEM. “Feeding” (F_2,27_ = 1.21), “drinking” (F_2,27_ = 2.93), “standing” (F_2,27_ = 1.29), “running” (F_2,26_ = 0.37), “preening” (F_2,27_ = 2.63) and “aggression” (F_2,26_ = 1.71) were not affected by density. “Sitting” and “perching” were affected by age×density (Figs. 1 & 2) and “disturbance” by barrier×density (Fig. 3).

2 Mixed model repeated measures ANOVA, df = 2,27, with the exception of foraging with df = 2,26 as foraging means were pooled across weeks.

a Means within a row with different superscripts are significantly different (P<0.05) after Tukey's comparison.

### Activity

Barrier treatment did not affect the proportion of time that birds were observed sitting, though there was a significant interaction between density and age (F_8,108_ = 3.57, P = 0.001; Figure 1), mostly due to a high proportion of sitting during week 5 in the 18 birds/m^2^ treatment compared to the 8 birds/m^2^ treatment (P = 0.015). In contrast, the proportion of observations of standing was not affected by density treatment but was by barrier such that birds in control pens spent more time standing compared to individuals in simple and complex barrier pens (F_2,27_ = 15.68, P<0.0001; Table 3). Age effect was also present: birds stood least frequently during week 2 and most frequently during week 4 (F_4,108_ = 26.19, P<0.0001; Table 2).

Proportion of walking was highest at the lowest density (F_2,27_ = 6.45, P = 0.005; Table 4) and at younger ages (F_4,108_ = 8.23, P<0.0001; Table 2) but was not affected by barrier treatment. Contrary to the effect on walking, the proportion of time that birds were observed running was not affected by density, though barrier treatment had an effect (F_2,26_ = 4.17, P = 0.027; Table 3) such that birds reared with simple barriers were observed running less often than birds in the control treatment (P = 0.022).

On the other hand, perching frequency was not affected by barrier type, though a clear density by age interaction effect was detected (F_8,72_ = 5.09, P<0.0001; Figure 2). The general trend for all density treatments was for perching to rise during the first few weeks of observation, peak at week 4, and decline thereafter. However, perching in the 18 birds/m^2^ treatment declined earlier than perching in the 8 birds/m^2^ pens (between 4 and 5 weeks of age, P = 0.041).

The proportion of observed preening was not influenced by either barrier or density treatment but was affected by age (F_4,108_ = 13.44, P<0.0001, Table 2), with a peak in preening observed at 3 weeks.

#### Aggression and Disturbances

The proportion of aggressive interactions was unaffected by density. However, birds in the simple and particularly complex barrier treatments experienced lower aggression as compared to controls (F_2,26_ = 12.30, P = 0.0002; Table 3). Rate of disturbances was affected by an interaction between barrier and density treatment (F_4,26_ = 3.28, P = 0.027; Figure 3). Disturbance frequency was highest at all densities for the control treatment compared to both barrier treatments and generally increased as density rose.

#### Use of space

The presence of barriers affected bird distribution (F_2,27_ = 8.46, P = 0.001; Table 3) such that use of the central area was more frequent when simple barriers were added to pens (P<0.0001 compared to control). Birds' occupation of the central area was also affected by density (F_2,27_ = 5.70, P = 0.009; Table 4) and was greatest in the 8 birds/m^2^ treatment compared to the 13 birds/m^2^ (P = 0.016) and 18 birds/m^2^ treatment (P = 0.021). Use of space was not affected by age.

## Discussion

Increasing environmental complexity in the form of barrier perches had clear benefits for broilers, not only by providing behavioral opportunities in the form of perching, but also by controlling aggressive interactions and reducing disturbances, especially at higher rearing densities.

In contrast with previous studies for modern broiler strains [21]–[23], we found that birds in either simple or complex barrier treatments allocated a substantial portion of time toward perching. Perching was observed nearly one-quarter of the time in the lowest density treatment during the fourth week of age, demonstrating that broilers have retained the ability and motivation to perch so long as they are provided with suitable environmental enrichment [20]. It is likely that the low height of the barrier perches used in this study translated into greater accessibility and thus more frequent use. Nevertheless, the decline in perching frequency with age and density was similar to the abovementioned studies.

In addition to perching, general activity – shown in high levels of walking and a reduction in sitting – peaked in week four. These results are consistent with earlier research [19], [21], [23]. The lower proportion of standing found in this study compared to prior work [19] can probably be explained by the higher rates of perching through the growth period. The consistency of results across studies related to the decline in activity with age suggests that rapid growth rates may influence broiler activity [5], [29], [30].

Although behaviors such as foraging, sitting and preening were unaffected by barrier treatment, barriers did influence appetitive behaviors. Lower feeding frequencies were observed in the complex barrier treatment compared to controls, in contrast to the lack of effects reported on wooden barriers in the past [19]. The additional arms of the complex barriers may explain this discrepancy, as resource access may be limited when barrier structure becomes too intricate. Differences in resource accessibility may also explain why drinking was more frequent in the control vs. simple barrier treatment, a finding that confirms the results of earlier barrier work [19]. However, given the lack of differences in feed conversion or final body weights reported in our companion study [24], it seems that birds may have adjusted their feeding strategies to meet the demands of navigating through their environment; thus, these results should not prompt much concern.

In addition to encouraging desirable behaviors, our findings suggest that barrier perches may have been an effective tool to minimize behaviors that have a strong negative impact on the welfare and performance of broiler chickens. Both aggressive interactions and disturbances were at least halved when barriers were added, and in one instance (complex barrier treatment), aggression was almost eradicated (See Table 3). It was recently demonstrated that providing visual barriers (tin sheets and straw bales) reduces aggression in breeding rink-necked pheasants, which could be explained by a number of factors including reduction in visual horizon, increased escape opportunities, or more even bird distribution in the pen [31]. In our study, the decrease in disturbances and aggression in the enriched environments may be explained by how the barriers affected birds' spatial distributions: their attraction as perching furniture along with the protection offered by the barrier edges may have dispersed resting areas throughout the pen [13]. This is supported by the fact that use of the central pen area increased when barriers were added to the pens (though not significantly so for the complex treatment) in comparison with control pens. Further, since aggression between broilers occurs mainly within the open areas of an enclosed environment [10], [15], barrier perches may have reduced the occurrence of such displays by breaking up this open space. In support of this theory, running (a behavior often performed in conjunction with aggressive displays) occurred most often in control pens, where birds did not have to navigate among barriers.

In this study we found a clear increase in disturbance frequency with higher densities, especially in the increase from 13 to 18 birds/m^2^. Similar effects have been previously observed [8], [10], [32] and can be explained by more chickens searching for resting locations along the wall [7], [10]. The welfare consequences of high disturbance rates are important, as disturbances interrupt resting time [10] and compromise body integrity due to a higher incidence of scratches inflicted by birds traveling through the resting group [33], [34]. We provided clear evidence that disturbances can be effectively managed by adding barrier perches, as rates with either barrier were lower than controls across all experimental densities. By reducing potential competition for prime resting locations around peripheral walls, barrier perches may have mitigated the negative repercussions of high density on disturbance rate.

In addition to the adverse effects on disturbances, high densities discouraged active behaviors and decreased use of the pen central space. The reduction in foraging and walking and the increase in sitting as density increased from 13 to 18 birds/m^2^, especially as birds aged, may relate to a reduction in floor space and subsequent opportunity to walk and forage [8], [34]. More specifically, the observed decline could be due to how density constrained the birds' movement in the available space [9] or simply by the fact that the allotted space became inadequate for birds to perform active locomotive behaviors as they grew [34]. We have shown elsewhere that footpad lesions are more serious at higher densities [24], so it is also possible that lesions (or other lameness issues) contributed to more frequent sitting earlier in life. In respect to the observed effects on foraging, birds may have been discouraged as the litter quality deteriorated and became more compacted in the high-density environments [35].

The decline in perch use at higher densities in this study contrasts with earlier work with perches and broilers [23]. As birds did use the barrier perches more frequently overall, it is possible that the total available perching space in our study limited the opportunity for some birds to perch at higher densities, particularly as they grew larger in size. Accounting for the types and number of barriers in the pens, and if each bird occupied approximately 15 cm of perch space, this would allow about 6.7 birds to simultaneously occupy each simple barrier perch (20 birds total in a simple barrier treatment pen), and 10.7 birds on a complex barrier perch (28 birds total in a complex barrier treatment pen). We did not observe these levels of saturation; therefore, additional barrier space may not have reduced the burden of high density more than what we found, though it is difficult to make this conclusion without further research. It is possible that high density could have influenced the results in another way, perhaps simply by hindering access to the barriers.

In summary, in this study we provided further evidence of the detrimental effects of increasing density, as attested by suppression of activity levels, increased disturbances and decreased use of perches and central areas. We also provide evidence that barrier perches, and more specifically, simple barrier perches, can be an effective tool to improve broiler welfare by (1) encouraging a broader behavioral repertoire that translated into increased activity levels and decreased aggression and disturbances and by (2) promoting improved use of space by increasing bird dispersion for resting. Based on the results of this and our companion study [24], we conclude that creating a more complex environment by using simple barrier perches is an advantageous, cheap strategy for producers to improve broiler health and welfare.
